# Microbial Upcycling of Depolymerized Lignin into Value-Added Chemicals

**DOI:** 10.34133/bdr.0027

**Published:** 2024-01-22

**Authors:** Yang Zhang, Cheng Cheng, Bixia Fu, Teng Long, Ning He, Jianqiang Fan, Zheyong Xue, Anqi Chen, Jifeng Yuan

**Affiliations:** ^1^ School of Life Sciences, Faculty of Medicine and Life Sciences, Xiamen University, Fujian 361102, China.; ^2^ Technology Center, China Tobacco Fujian Industrial Co. Ltd., Xiamen 361000, Fujian, China.; ^3^ College of Chemistry and Chemical Engineering, Xiamen University, Fujian 361005, China.; ^4^ Ministry of Education, Key Laboratory of Saline-alkali Vegetation Ecology Restoration, Northeast Forestry University, Heilongjiang 150040, China.; ^5^ Science Center for Future Foods, Jiangnan University, Jiangsu 214122, China.

## Abstract

Lignin is one of the most widespread organic compounds found on earth, boasting a wealth of aromatic molecules. The use of lignin feedstock for biochemical productions is of great importance for achieving “carbon neutrality.” In recent years, a strategy for lignin valorization known as the “bio-funnel” has been proposed as a means to generate a variety of commercially valuable chemicals from lignin-derived compounds. The implementation of biocatalysis and metabolic engineering techniques has substantially advanced the biotransformation of depolymerized lignin into chemicals and materials within the supply chain. In this review, we present an overview of the latest advancements in microbial upcycling of depolymerized lignin into value-added chemicals. Besides, the review provides insights into the problems facing current biological lignin valorization while proposing further research directions to improve these technologies for the extensive accomplishment of the lignin upcycling.

## Introduction

Due to the industrial reliance on fossil fuels along with the increasing concern about environmental pollution and human health, there has been a soaring interest in developing a low-carbon society. The efficient utilization of biomass resources is of great significance in contributing to the achievement of “carbon peaking and carbon neutrality.” Lignin, a natural amorphous polymer, is composed of three phenylpropane units (coniferyl, *p*-coumaryl, and sinapyl alcohols) through free radical coupling, forming the relevant guaiacyl (G), *p*-hydroxyphenyl (H), and syringyl (S) monomers. These three monomers then join together through a range of C-O and C-C bonds, for instance, α-*O*-4, β-*O*-4, and 4-*O*-5 bonds, resulting in the formation of complex lignin structures [1]. It is estimated that the worldwide pulp and paper industry produces roughly 70 million tons of lignin-based byproducts per year, while the total annual lignin production is approximately 34 billion tons [2].

Although the techniques for transforming and utilizing cellulose and hemicellulose are fairly advanced [3,4], the intricate and diversified composition of lignin poses a challenge for its effective utilization. The bulk of lignin is discarded as wastes in the paper and pulp industry or burned as fuels. Furthermore, recent economic analyses have demonstrated that producing value-added chemicals from lignin is at least 10 times more lucrative and less detrimental to the environment than using it for energy generation [5–7]. Given this, it is essential to explore novel approaches to transform lignin into value-added chemicals. At present, most researches on the transformation of lignin into chemicals rely on the two-stage strategy, which involves decomposing the polymer before refining the degraded molecules. In the decomposition step, the macromolecular lignin can be broken down into lower-mass phenolic compounds through various pretreatment strategies and depolymerization techniques including oxidative depolymerization, reductive depolymerization, thermal depolymerization, and chemical modification [8]. During the refining step, these lower-mass phenolic compounds can be further transformed into a range of more specialized and high-value chemicals.

Various phenolic compounds with rich functional groups can be obtained from diverse lignin sources through different extraction and fractionation methods as well as depolymerization strategies. Unfortunately, the majority of these compounds usually have no immediate practical use and require additional processing to be transformed into fuels, chemicals, and materials. Because of the inconsistent chemical composition of these products, different downstream operations must be devised to obtain the sought-after product. Recently, more microbes with the ability to degrade lignin-derived aromatics have been identified, and their degrading enzymes have been elucidated, which provide an essential molecular basis for engineering microbes to maximize lignin utilization. For instance, certain bacterial and fungal species such as *Pseudomonas putida* [9], *Rhodococcus opacus* [10], *Rhodopseudomonas palustris* [11], *Phanerochaete chrysosporium* [12], and *Sphingomonas* [13] have been identified as effective lignin-upcycling strains. These organisms possess a range of enzymes and cofactors to directly utilize pretreated lignin or depolymerized aromatic compounds. In addition, a number of studies have revealed that by expressing heterologous enzymes in the model microorganism *Escherichia coli*, the transformation of lignin-based platform compounds into more valuable end products can be realized.

**Fig. 1.**
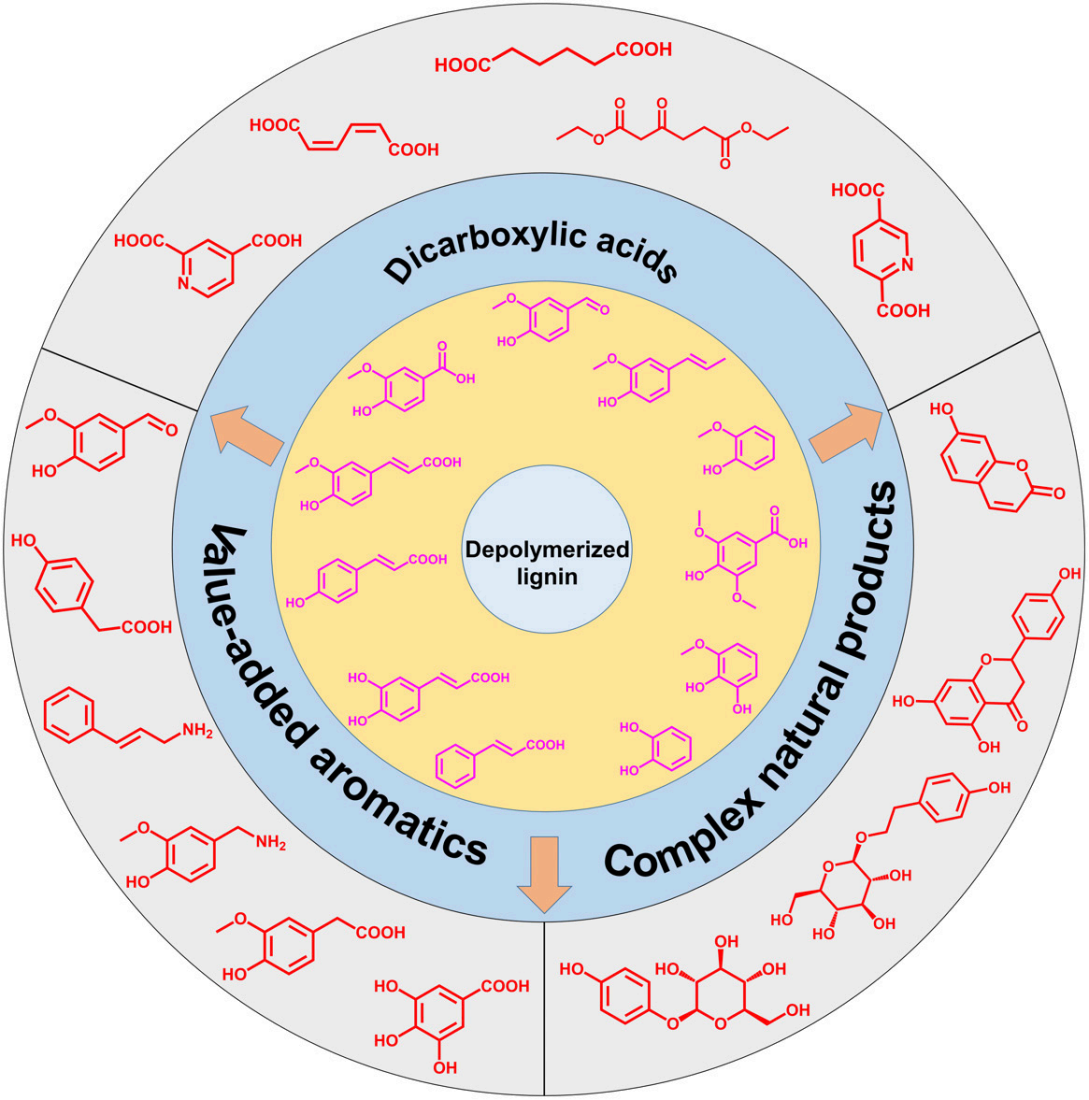
Schematic representation of microbial upcycling of lignin-derived compounds into value-added chemicals. The lignin can be depolymerized through a variety of methods to produce heterogeneous mixtures consisting of various aromatic monomers, dimers, and oligomers. Some microorganisms can metabolize lignin-derived aromatic compounds by utilizing their powerful aromatic degradation pathways, resulting in various value-added products. Engineered microbes with heterologous enzymes can be used to functionalize lignin-derived aromatic compounds into other value-added chemicals.

This review presents an overview and outlook of extensive investigations into the lignin-to-product system, focusing primarily on the bioprocessing of depolymerized lignin into end products. We summarize the current progress in biological approaches for lignin valorization using microbes. Considering the potential of biosynthesis from lignin-derived feedstocks using diverse microorganisms (Fig. 1), we delve into the latest progress in the downstream bioprocessing of depolymerized lignin into dicarboxylic acids, value-added aromatics, and complex natural products. Moreover, this review seeks to promote the sustainable utilization of lignin by proposing strategies from biocatalysis and metabolic engineering perspectives, thus integrating this valuable “biowaste” into chemical supply chains through the synthesis of value-added products.

## Recent Advances of Dicarboxylic Acids from Lignin-Depolymerized Compounds

### Biosynthesis of *cis*, *cis*-muconic acid

*cis*, *cis*-Muconic acid (ccMA), a common dicarboxylic acid, serves as a key precursor for producing adipic acid, which is a major source of raw material for fibers and plastics. The global market of ccMA has been evaluated to be worth over $22 billion. Presently, most ccMA is still generated by chemical synthesis from petroleum-based raw materials [7]. The emergence of synthetic biology offers an alternative approach, enabling the production of ccMA from sustainable carbon sources. Various studies have exhibited that a variety of bacteria can transform catechol into ccMA when exposed to catechol 1,2-dioxygenase. Additionally, lignin-derived compounds such as benzene, phenol, toluene, benzoate, and cinnamic acid can also be converted to ccMA through catechol-mediated reactions [3]. In lignin valorization, protocatechuic acid is a node compound that can be transformed from *p*-coumaric acid, ferulic acid, and vanillin, which are also essential products in lignin valorization. Vardon et al. [14] attempted to identify the key enzymes to bridge the protocatechuic acid and catechol branches. They engineered the strain *P. putida* containing a productive artificial synthesis pathway that effectively converts a selection of lignin-derived aromatic compounds into ccMA (Table 1) while avoiding further breakdown of protocatechuic acid.

**Table 1.** Recent advances of dicarboxylic acids from lignin-depolymerized compounds

Strain	Main strategies	Substrates	Product	References
*P. putida* KT2440-CJ103	Express *aroY* gene; delete *pcaHG* and *catB* genes; apply dissolved oxygen static fed-batch fermentation	*p*-Coumaric acid	ccMA (13.5 g/l)	[14]
*P. putida* KT2440-CJ103	Introduce *aroY* gene; delete *pcaHG* and *catB* genes	Alkaline pre-treated liquor	ccMA (0.7 g/l)	[14]
*P. putida* KT2440-CJ242	Perform a fed-batch fermentation using high pH solution with *p*-coumaric acid as feeding solution	*p*-Coumaric acid	ccMA (50 g/l)	[15]
*P. putida* KT2440-CJ242	Perform a constant fed-batch fermentation process	Base-catalyzed depolymerized lignin	ccMA (3.7 g/l)	[15]
*P. putida* MA-9	Construct a strain with high tolerance to catechol; enhance catechol 1,2-dioxygenase expression levels	Softwood lignin hydrolysate	ccMA (13 g/l)	[30]
*Amycolatopsis* sp. ATCC 39116	Delete two putative *catB *genes; perform a fed-batch fermentation	Guaiacol	ccMA (3.1 g/l)	[16]
*Amycolatopsis* sp. ATCC 39116	Delete two putative *catB* genes; perform a fed-batchfermentation	Softwood lignin hydrolysate	ccMA (255.8 mg/l)	[16]
*C. glutamicum* MA-2	Delete *catB* gene; express *catA* gene; apply a fed-batch fermentation process	Catechol	ccMA (85 g/l)	[17]
*C. glutamicum* MA-2	Delete *catB* gene; express *catA* gene; apply a fed-batch fermentation process	Lignin hydrolysate	ccMA (1.8 g/l)	[17]
*Sphingobium* sp. SME257/pTS084	Use G-lignin components for ccMA production and S-lignin components for cell growth	Hardwood lignin hydrolysate	ccMA (26.8 mg/l)	[18]
Engineered *E. coli*	Coexpress four genes of *vdh*, *desA*, *catA*, and *aroY*	Vanillin	ccMA (341 mg/l)	[7]
Engineered *E. coli*	Coexpress genes of *vdh*, *vanAB*, *catA*, *aroY*, and *kpdB*	Vanillin	ccMA (-)	[22]
Engineered *E. coli*	Overexpress *ado*, *hfd1*, *vanAB*, *gdc*, and *catA* genes; apply two-step catalysis and fed-batch process	Isoeugenol	ccMA (1.42 g/l)	[23]
Engineered *E. coli*	Overexpress *vanAB*, *gdc*, and *catA* genes; apply a fed-batch process	Vanillic acid	ccMA (6.22 g/l)	[23]
Engineered *P. putida*	Overexpress *pcalJ*, *paaH*, *paaF*, *and ter* genes; delete *pcaF*, *paaJ* genes; perform a constant fed-batch fermentation process	*p*-Coumaric acid and ferulic acid	Adipic acid (2.5 g/l)	[28]
Engineered *E. coli*	Introduce *gcoAB*, *catA*, and *bcER* genes	Guaiacol	Adipic acid (670 mg/l)	[29]
Engineered *R. jostii*	Delete *pcaHG* and heterologously express *praA*	Wheat straw hydrolysate	Pyridine-2,5-dicarboxylic acid (287 mg/l)	[31]
Engineered *R. jostii*	Integrate *ligAB* into *pcaHG* and overexpress *dyp2*	Commercially available soda lignin	Pyridine-2,4-dicarboxylic acid (240 mg/l)	[31]
Engineered *P. putida*	Delete *pcaJ*; heterologously express *ligV*; overexpress *vanAB*, *pcaHG*, and *pcaBCD*	Vanillin or vanillate	β-Ketoadipate (23 g/l)	[32]

Investigations have also been extensively conducted to optimize the ccMA synthetic pathway in microbial cell factories. Strategies such as metabolic regulation, steady batch feeding, and high-pH feeding were tested in a recombinant *P. putida* with the overexpression of *catA* on *catRBC* locus, the overexpression of *aroY* and *ecdBD* on *pcaHG*, and the deletion of *crc*, and the titers of ccMA from *p*-coumaric acid and alkali-mediated lignin hydrolysate were significantly enhanced, reaching up to 50 g/l and 3.7 g/l, respectively [15]. Similarly, *Amycolatopsis* sp., with the absence of two ccMA cycloisomerases, produced 3.1 g/l and 255.8 mg/l ccMA from guaiacol and softwood lignin hydrolysate, respectively [16]. In another case, the mutant of *Corynebacterium glutamicum* with the deletion of *catB* and the overexpression of *catA*, known for its robust tolerance to lignin-derived aromatic hydrocarbons, accumulated 85 g/l and 1.8 g/l ccMA from catechol and lignin hydrolysate, respectively [17]. Most of these studies indicated that extra glucose or organic acids were required during fermentation to sustain cell growth. However, Sonoki et al. [18] created a recombinant of *Sphingobium* sp., which is able to utilize S-lignin derivatives for cell growth and G-lignin derivatives for ccMA synthesis, accomplishing 26.8 mg/l ccMA from hardwood lignin without the need of additional glucose. Recently, *E. coli* has been harnessed to exploit lignin-based aromatics owing to its rapid growth rate, well-established genetic background, and accessible genetic tools [19–21]. The titers of 100 to 341 mg/l ccMA were obtained from vanillin based on different synthetic biology strategies (Table 1) [7,22]. In addition, an engineered *E. coli* whole-cell biocatalytic system was developed to transform lignin derivatives, such as isoeugenol and vanillic acid, into ccMA, resulting in productions of 1.42 g/l and 6.22 g/l, respectively [23].

### Biosynthesis of adipic acid

Adipic acid, as one of the most commercially important dicarboxylic acids, holds a market value of $6.3 billion with a production of 2.6 million tons per year and is estimated with an increasing global demand of 3 to 3.5% each year [24,25]. The majority of adipic acid is utilized as a comonomer in polymer production, specifically in combination with 1,6-hexanediamine to generate nylon-6,6, one of the most broadly applied thermoplastics [25]. Its commercial production mainly relies on the oxidation of petrochemically derived cyclohexanone/cyclohexanol by using concentrated nitric acid in a metal-catalyzed process [26], which significantly consumes and emits nitrous oxide, a greenhouse gas of 300 times more potent than CO_2_. Given the unsustainability of this chemical approach, multiple studies have been conducted to explore an eco-friendly and sustainable route for adipic acid synthesis [25,27]. Niu et al. [28] overexpressed *pcaIJ*, *paaHF*, and *ter* genes and deleted *pcaF* and *paaJ* genes in *P. putida*, enabling the engineered strains to produce adipic acid from common aromatic hydrocarbons with a titer of 0.76 g/l under shake flask conditions, and a titer of 17.4 g/l under fermenter conditions. Suitor et al. [29] developed a whole-cell biocatalytic system in *E. coli* by introducing *gcoAB*, *catA*, and *bcER* genes, achieving a final titer of 670 mg/l adipic acid from guaiacol in one-pot biotransformation. Besides, techniques such as adipic acid catalytic hydrogenation and polymerization have demonstrated the feasibility of transforming lignin into nylon-6,6 [30].

In addition to ccMA and adipic acid, several studies showed the biosynthesis of pyridine-dicarboxylic acids and beta-ketoadipate. Spence et al. [31] engineered *Rhodococcus jostii* strain by disrupting *pcaHG* and introducing heterologous *praA* to produce 287 mg/l pyridine-2,5-dicarboxylic acid from wheat straw hydrolysate [31]. They also engineered another recombinant by integrating *ligAB* on *pcaHG* site and overexpressing *dyp2* to produce 240 mg/l pyridine-2,4-dicarboxylic acid from commercially available soda lignin. Suzuki et al. [32] inactivated *pcaJ*, heterologously expressed *ligV*, and overexpressed *vanAB*, *pcaHG*, and *pcaBCD* in *P. putida* to obtain 23 g/l β-ketoadipate from vanillin or vanillate with more than 93% conversion and 0.88 g/l β-ketoadipate from softwood lignin extracts.

## Recent Advances of Value-Added Aromatics from Lignin-Depolymerized Compounds

### Biosynthesis of vanillin

Vanillin can be directly obtained from lignin depolymerization and lignin-derived compounds by biological means. Despite that the chemical decomposition of lignin being the conventional approach for industrial vanillin production [33], there is still an increasing interest in developing biological approaches for vanillin production. Ferulic acid, one of the most plentiful aromatic components in lignocellulosic biomass, can be efficiently generated by thermochemical depolymerization and enzymatic hydrolysis [34]. Studies have exhibited that vanillin can be synthesized from ferulic acid via nonoxidative decarboxylation and coenzyme A (CoA)-dependent deacetylation pathways [3]. In the nonoxidative decarboxylation pathway, ferulic acid gets converted into 4-vinyl guaiacol by decarboxylase, which is further transformed into vanillin by oxygenase [35,36]. In the CoA-dependent deacetylation pathway, ferulic acid is converted to feruloyl-CoA by feruloyl-CoA synthetase (FCS), which is then processed by enoyl-CoA hydratase/aldolase (ECH) into 4-hxdroxy-3-methoxyphenyl-β-hydroxypropionyl-CoA, and eventually into vanillin [37,38]. More recently, Ni et al. [39] formulated a coenzyme-free biocatalyst using phenolic acid decarboxylase (Pad) and aromatic dioxygenase (Ado), and achieved an efficient transformation of lignin-derived phenolic acid compounds into aldehydes, leading to a titer of 15.22 g/l vanillin from ferulic acid.

### Biosynthesis of gallic acid

Gallic acid, a natural polyphenolic compound, demonstrates extensive applications in various fields, including chemical, pharmaceutical, food, and dye productions [40]. Typically, gallic acid is generated through acid or alkali hydrolysis of tannins, microbial fermentation of tannins, or tannin hydrolysis utilizing highly efficient tannases [41,42]. These methods are limited by the dependency on tannins as the primary input material. Hence, researchers have been seeking out alternative ways to effectively generate gallic acid from more sustainable resources. Eschrich et al. [43] reported the first successful transformation of 3,4-dihydroxybenzoate into gallic acid utilizing the mutated hydroxybenzoate hydroxylase (PobA^Y385F^). Later, Kambourakis et al. [44] and Brückner et al. [45] synthesized 20 g/l gallic acid from glucose by the engineered *E. coli* with the integration of *aroZ* into *serA* locus and the overexpression of *aroF^FBR^* and *pobA^Y385F^* and 682 mg/l gallic acid from glucose by the engineered *Saccharomyces cerevisiae* with enhanced shikimate pathway and the overexpression of *aroZ* and *pobA^Y385F^*. Additionally, Wu et al. [7] demonstrated the catalysis of lignin-derived syringate to generate 59.6 mg/l gallate in the presence of syringate *O*-demethylase (DesA) and 3-*O*-methylgallate-*O*-demethylase (LigM). To further explore the production of gallic acid from inexpensive lignin-derived aromatics, Fu et al. [46] built two novel biocatalytic pathways containing FCS-ECH-HFD1 (aldehyde dehydrogenase)-VanAB (vanillic acid *O*-demethylase)-PobA^Y385F^ or HpaBC (two-component flavin-dependent monooxygenase)-FCS-ECH-HFD1-PobA^Y385F^ in *E. coli* to investigate the potential of transforming ferulic acid and *p*-coumaric acid into gallic acid, ultimately achieving a maximum production of 3.33 g/l gallic acid. Cai et al. [47] deleted the endogenous degradation pathways of protocatechuic acid and gallic acid, and introduced DesA, LigM, and PobA^Y385F/T294A^ in *R. opacus*, yielding 0.42 g/l gallic acid from alkaline-pretreated lignin stream.

### Biosynthesis of aromatic amines

Aromatic amines are extensively employed in the chemical industry for the manufacture of various pharmaceuticals, agrochemicals, cleaning supplies, and personal care products [48]. Hence, an increasing number of studies are embracing lignin-derived aromatic hydrocarbons as potential feedstocks to streamline the biosynthesis of aromatic amines (Table 2). Vanillylamine serves as a crucial intermediate for the production of natural capsaicinoids and their analogs [49], which are pivotal in the manufacture of pharmaceuticals, environment-friendly biological pesticides, and food additives [50,51]. Manfrao-Netto et al. [52] revealed that overexpressing amine aminotransferases (TAs) in *P. putida* enhanced vanillylamine production to 174 mg/l from vanillin. In another study, Du et al. [53] assessed eight potential ω-transaminase substrates and found that vanillin was the most effective for vanillylamine production, achieving 5.5 g/l vanillylamine with (*S*)-α-methylbenzylamine (*S*-MBA) as the amino donor. However, since *S*-MBA cannot be recovered in the enzymatic system, acetophenone was produced as a by-product. Fu et al. [54] designed a whole-cell biocatalysis system in *E. coli* by separately introducing FCS, ECH, TA, and alanine dehydrogenase (AlaDH), or carboxylic acid reductase (CAR), phosphopantetheine transferase (PPTase), TA, and AlaDH, enabling the conversion of lignin-derived ferulic acid and vanillic acid into vanillylamine with an optimum titer of 3.64 g/l through a one-step procedure using ammonium salts as amino donors. Cinnamylamine, an aromatic amine derived from _L_-phenylalanine, is applied in the production of various bioactive compounds with therapeutic properties [55–57]. While the predominant synthesis of cinnamylamine remains chemical means [58,59], recent studies have focused on its biological production. Wang et al. [60] identified a biocatalytic pathway containing 4-coumarate: CoA ligase (4CL), cinnamyl-CoA reductase (CCR), CAR, PPTase, and ωTA for the cinnamylamine biosynthesis in *E. coli*, reaching a titer of 523.15 mg/l by screening key enzymes and optimizing fermentation substrates and cofactors.

**Table 2.** Recent advances of fine aromatic compounds from lignin-depolymerized compounds

Strain	Main strategies	Substrates	Product	References
Engineered *E. coli*	Express *pad* and *ado* genes; apply the coenzyme-free biocatalyst LV (lignin-to-valuables) and employ chloroform/water system	Ferulic acid	Vanillin (15.22 g/l)	[39]
Engineered *E. coli*	Coexpress *pad*, *styAB*, *styC*, *adh*, *par1* with *yqhD* genes	*p*-Coumaric acid	Tyrosol (2.05 g/l)	[68]
Engineered *E. coli*	Introduce *pad*, *styAB*, *styC*, *adh*, *par1*, *yqhD*, and *hpaBC* genes	*p*-Coumaric acid	Hydroxytyrosol (1.83 g/l)	[68]
Engineered *E. coli*	Express *pad*, *styAB*, *styC*, *adh*, *par1*, and *yqhD* genes	Ferulic acid	Homovanillyl alcohol (1.19 g/l)	[68]
Engineered *E. coli*	Introduce *pad*, *styAB*, *styC*, and *feaB* genes	*p*-Coumaric acid	4-Hydroxy-phenylacetic acid (2.08 g/l)	[61]
Engineered *E. coli*	Express *pad*, *styAB*, *styC*, *feaB*, and *hpaBC* genes	*p*-Coumaric acid	3,4-Dihydroxy-phenylacetic acid (2.27 g/l)	[61]
Engineered *E. coli*	Coexpress *pad*, *styAB*, and *styC* with *feaB* genes	Ferulic acid	Homovanillic acid (692 mg/l)	[61]
Engineered *E. coli*	Express *fdc1*, *smo*, *soi*, and *par* genes	*p*-Coumaric acid	Tyrosol (2.07 g/l)	[64]
Engineered *E. coli*	Coexpress *desA*, *ligM*,and *lpdc* genes	Syringic acid	Pyrogallol (7.3 mg/l); Gallic acid (18 mg/l)	[7]
Engineered *E. coli*	Integrate *aroZ* into *serA* locus and overexpress of *aroF^FBR^* and *pobA^Y385F^*	Glucose	Gallic acid (20 g/l)	[44]
Engineered *E. coli*	Introduce *fcs*, *ech*, *hfd1, hpaBC*, and *pobA^Y385F^* genes	*p*-Coumaric acid	Gallic acid (3.4 g/l)	[46]
Engineered *E. coli*	Introduce *fcs*, *ech*, *hfd1, vanAB*, and *pobA^Y385F^* genes	Ferulic acid	Gallic acid (3.33 g/l)	[46]
*P. putida* KT2440-GN442ΔPP_2426	Overexpress *cvTA* and *aladh* genes; delete *catA*, *vdh*, *ald*, and *bdh* genes; L-alanine and/or NH_4_Cl as the amine donor	Vanillin	Vanillylamine (174 mg/l)	[52]
Engineered *E. coli*	Express *cvTA* gene; (*S*)-α-methylbenzylamine as the amine donor	Vanillin	Vanillylamine (5.5 g/l)	[53]
Engineered *E. coli*	Introduce *fcs*, *ech*, *cvTA*, and *aladh* genes; NH_4_Cl as the amine donor	Ferulic acid	Vanillylamine (3.64 g/l)	[54]
Engineered *E. coli*	Introduce *CAR/PPTase*, *cvTA*, and *aladh* genes; NH_4_Cl as the amine donor	Vanillic acid	Vanillylamine (3.58 g/l)	[54]
Engineered *E. coli*	Coexpress *CAR/PPTase* with *cvTA* gene	Cinnamic acid	Cinnamylamine (523.15 mg/l)	[60]

### Biosynthesis of other phenolic acids

There has also been an extensive effort in converting lignin-derived compounds into high-value phenolic acids. For instance, Zhao et al. [61] constructed a one-pot biotransformation system in a reduced aromatic aldehyde reduction *E. coli* containing two routes: Pad-StyAB (styrene monooxygenase)-StyC (styrene oxide isomerase)-FeaB (phenylacetaldehyde dehydrogenase) and Pad-StyAB-StyC-FeaB-HpaBC (4-hydroxyphenylacetate 3-hydroxylase), producing 2.08 g/l 4-hydroxyphenylacetic acid, 2.27 g/l 3,4-dihydroxyphenylacetic acid, and 692 mg/l homovanillic acid from lignin-derived *p*-coumaric acid and ferulic acid. Austin et al. [11] regulated the endogenous degradation pathway of *R. palustris* to yield benzoic acid and *p*-hydroxybenzoic acid from corn stover hydrolysate pretreated by ammonia fiber expansion. Studies on the biosynthesis of tyrosol, another highly sought-after aromatic compound in food and pharmaceutical industries, predominantly utilizes glucose or tyrosine as starting materials [62,63]. To generate tyrosol from lignin derivatives, Lai et al. [64] reported a synthetic pathway in *E. coli*, including ferulic acid decarboxylase (FDC1), styrene monooxygenase (SMO), styrene oxide isomerase (SOI), and phenylacetaldehyde reductase (PAR), achieving the conversion of *p*-coumaric acid to 2.07 g/l tyrosol.

## Recent Advances of Complex Natural Products from Lignin-Depolymerized Compounds

Plant-derived natural phenolic compounds, such as phenolic glycosides, coumarins, stilbene (e.g., resveratrol, paclitaxel, and pallitol), and flavonoids (e.g., quercetin, genistein, and apigenin) are valued chemicals in pharmaceutical and cosmetic industries due to their pharmaceutical activities [65–67]. However, their complex structures make efficient synthesis challenging using conventional chemical approaches. To address this issue, Zhao et al. [68] developed three novel biocatalytic enzyme-cascade pathways to synthesize phenolic glycosides such as gastroside, arbutin, and salidroside, along with their derivatives from lignin-derived compounds (Table 3). Utilizing lignin-derived hydroxycinnamate (mainly ferulic acid and *p*-coumaric acid) to directly supply aromatic units, these cascade reactions demonstrated a remarkable 100-fold increase in the biosynthesis productivity compared to that of the glucose-based biosynthesis.

**Table 3.** Recent advances of complex natural products from lignin-depolymerized compounds

Strain	Main strategies	Substrates	Product	References
Engineered *E. coli*	Introduce *pad*, *ado* (or *fcs* and *ech*), *adh*, *par1*, and *ugt73b6^FS^* genes	*p*-Coumaric acid	Gastrodin (1.46 g/l)	[68]
Engineered *E. coli*	Express *fcs*, *ech*, *vdh*, *mnx1*, and *as* genes	*p*-Coumaric acid	Arbutin (3.05 g/l)	[68]
Engineered *E. coli*	Coexpress *pad*, *styAB*, *styC*, *adh*, *par1*, *yqhD* with *ugt85a1* genes	*p*-Coumaric acid	Salidroside (4.22 g/l)	[68]
Engineered *E. coli*	Introduce *4cl* and *f6’h2* genes	*p*-Coumaric acid	Umbelliferone (82.9 mg/l)	[71]
Engineered *E. coli*	Introduce *4cl* with *f6’h1* genes	Caffeic acid	Esculetin (52.3 mg/l)	[71]
Engineered *E. coli*	Introduce *4cl* and *f6’h2* genes	Ferulic acid	Scopoletin (79.5 mg/l)	[71]
Engineered *E. coli*	Coexpress *os4cl*, *ibf6’h, ats8’h* with *omt* genes	Ferulic acid	Fraxetin (175 μg/l)	[72]
Engineered*S. cerevisiae*	Introduce *4cl* and *f6’h1* genes	Lignin hydrolysate	Scopoletin (4.79 mg/l)	[73]
Engineered*C. glutamicum*	Express *4cl*, *sts*, and *romt* genes	*p*-Coumaric acid	Pterostilbene (42 mg/l)	[75]
Engineered*C. glutamicum*	Introduce *4cl*, *chs*, *chi*, *f3h*, and *fls* genes	*p*-Coumaric acid	Kaempferol (23 mg/l)	[75]
Engineered*C. glutamicum*	Introduce *4cl*, *chs*, *chi*, *f3h*, and *fls* genes	Caffeic acid	Quercetin (10 mg/l)	[75]
Engineered*S. venezuelae*	Heterologous expression of *4cl*, *chs*, *chi*, and *matBC* genes	*p*-Coumaric acid	Naringenin (35.6 mg/l)	[76]
Engineered*S. cerevisiae*	Expression of *4cl*, *chs*, and *chi* genes; a set of naive promoters was optimized	*p*-Coumaric acid	(2*S*)-naringenin (1.21 g/l)	[77]
Engineered *E. coli*	Coexpress *gcoAB* with *cueO* genes; apply a two-step one-pot strategy	3-Methoxy-catechol	Purpurogallin (46 mg/l)	[78]

Coumarins are generally categorized into four groups based on the 1,2-benzopyrone skeleton and the substituents present on the aromatic ring, namely, simple coumarins, furan coumarins, pyrano coumarins, and isoprenyl coumarins [69]. However, the commercial coumarins production typically relies on plant extraction. The development of synthetic biology promotes the exploration of biosynthetic approaches as an alternative to plant extraction in coumarins production. For instance, umbelliferone, a simple coumarin, can be generated from phenylalanine or tyrosine through phenylalanine (or tyrosine) ammonia lyase (PAL/TAL), cinnamate 4-hydroxylase (C4H), 4-cinnamic acid CoA ligase (4CL), and feruloyl CoA 6'-hydroxylase (F6'H) in the model microbe *E. coli* [70]. Recently, two common lignin-derived compounds, *p*-coumaric acid and ferulic acid, have been extensively studied as the substrates for coumarin production. Yang et al. [71] developed the synthetic pathway based on the cascade enzymes of TAL-4CL-F6'H to synthesize 82.9 mg/l umbelliferone from *p*-coumaric acid, 79.5 mg/l esculetin from caffeic acid, and 52.3 mg/l scopoletin from ferulic acid in *E. coli*. An et al. [72] harnessed the *E. coli* harboring 4CL, F6'H, *O*-methyltransferase (OMT), and scopoletin 8-hydroxylase to synthesize 175 μg/l fraxetin from ferulic acid. Zhao et al. [73] constructed a biocatalytic pathway containing HpaBC, OMT, and a fusion of 4CL-F6'H in the engineered *S. cerevisiae*, forming 4.79 mg/l scopoletin from lignin hydrolysate.

Certain flavonoids can be produced from lignin-derived compounds through processes involving acylation, Claisen condensation, cyclization, and hydroxylation [74]. The rapid development in microbial engineering has greatly facilitated the flavonoid biosynthesis from phenolic acids such as *p*-coumaric acid, caffeic acid, and ferulic acid. Kallscheuer et al. [75] engineered *C. glutamicum* strains by integrating 4CL to synthesize stilbenoids and flavonoids from *p*-coumaric acid and caffeic acid, eventually leading to the productions of 42 mg/l pterostilbene, 23 mg/l kaempferol, and 10 mg/l quercetin through the heterologous expression of OMT-STS (stilbene synthase) or CHS (chalcone synthase)-CHI (chalcone isomerase)-F3H (flavanone 3-hydroxylase)-FLS (flavonol synthase). Park et al. [76] engineered *Streptomyces venezuelae* to express 4CL, CHS, CHI, and MatBC (malonyl-CoA synthetase and dicarboxylate carrier protein), leading to a naringenin production of 35.6 mg/l from *p*-coumaric acid. Furthermore, Gao et al. [77] fine-tuned a group of promoters to regulate heterologous gene expressions including 4CL, CHS, and CHI, resulting in a titer of 1.21 g/l (2*S*)-naringenin from *p*-coumaric acid in *S. cerevisiae*. In addition, Zhang et al. [78] designed an *E. coli* whole-cell transformation using the laccases CueO and P450 GcoAB identified from *E. coli*, enabling the conversion of 5 mM lignin-derived 3-methoxycatechol into 0.67 g/l purpurogallin, an aglycone natural product with pharmaceutical value.

### Microbial direct processing of lignin or lignocellulosic biomass

To date, establishing biosynthetic pathways to upcycle the depolymerized lignin in model microbes is not relatively difficult. To simplify the bioproduction process, it is ideal to directly utilize lignocellulosic hydrolysates, which can provide additional carbohydrates such as glucose and xylose to support the cell growth. The direct microbial decomposition of lignocellulose for producing a variety of molecules is of particular interest, as it does not require expensive metal catalysts and difficult catalyst separation. Lignin-degrading bacteria are widely existing in natural lignin-rich environments, such as fallen leaves, decomposed forests, pulp mill sludge, soil, and activated sludge [79–81]. For instance, the wood-feeding termites play an important role in the natural carbon cycle, with most lignocellulosic materials being digested by the termite’s digestive system [82]. Therefore, the termite gut is a rich source for lignin-degrading bacteria, where some lignin degraders have been successfully isolated [83,84]. In addition, some endophytes possess a strong ability to degrade lignocellulose [85].

**Fig. 2.**
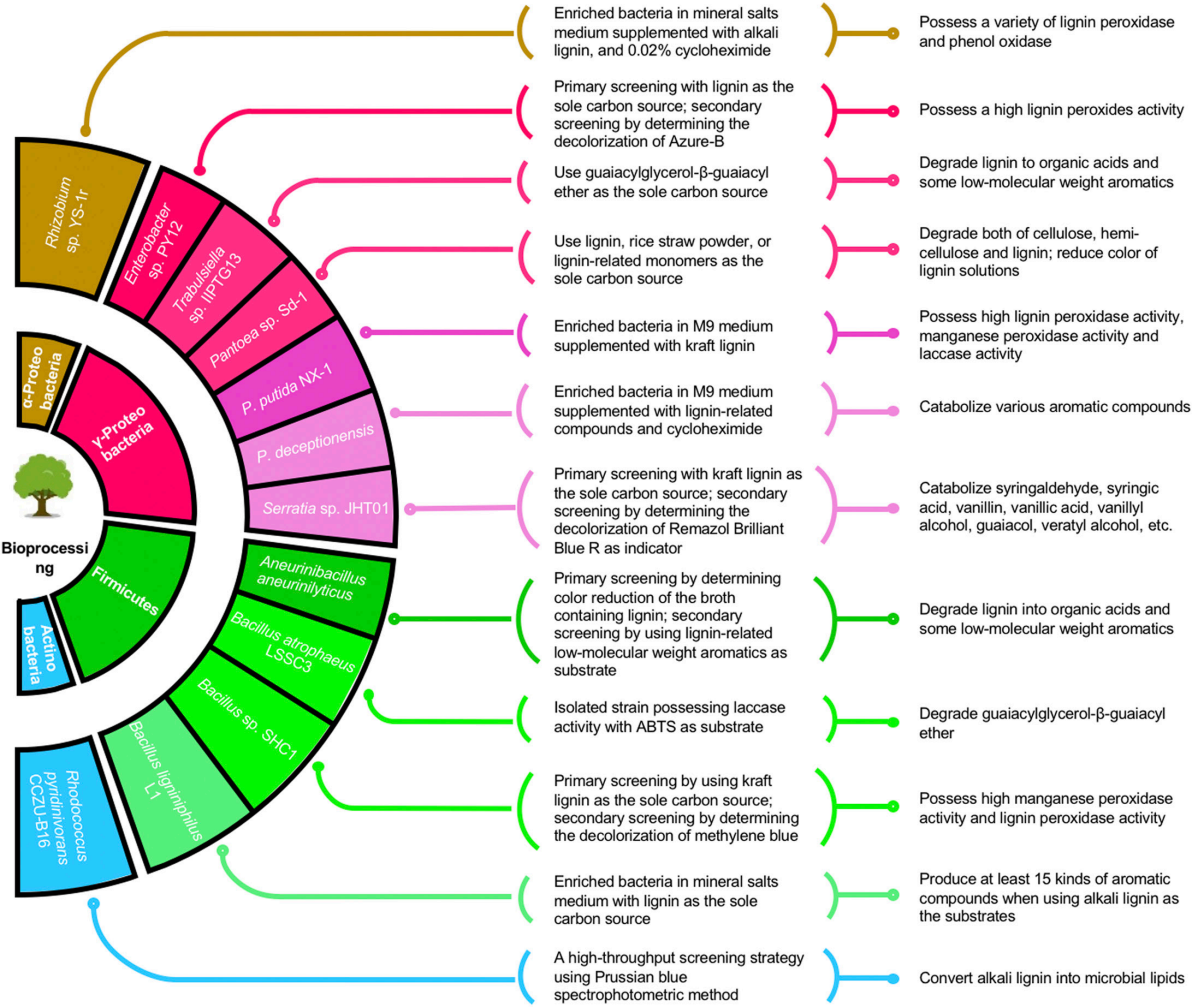
Microbial direct bioprocessing of lignin or lignocellulose into value-added chemicals through the biological funnel pathway. The lignin-degrading bacteria and their properties are summarized.

As summarized in Fig. 2, the identified lignin-degrading bacteria mainly belong to the phyla of Actinobacteria, Proteobacteria, Firmicutes, and Archaea. The more genotypic and phenotypic information related to lignin-degrading bacteria has been described in the literature [13,86]. Lignin degradation pathways and associated enzymes in some of bacteria have been characterized, including pathways associated with the depolymerization of phenolic and nonphenolic lignin polymers, catalytic oxidation and hydroxylation reactions, demethylation reactions, and lignin-based aromatic ring opening reactions [87–93]. Currently, some of these bacteria have been used for the pretreatment of lignocellulosic biomass from the pulp and paper industry [94–97]. Although a variety of lignin-degrading bacteria have been identified using the selective cultivation, their lignin-degrading abilities are still not fully elucidated. More efforts are still required to decipher lignin degradation pathways before an efficient consolidated process can be implemented for lignin valorization.

## Concluding Remarks

Lignin, the second-most abundant component of plants, has been shown to be a promising natural resource. Although upcycling the untreated lignin into chemicals by bacteria presents a challenging bottleneck in lignin valorization, significant progress has been made in microbial conversion using the depolymerized lignin. Future research should explore the potential for industrial-level lignin valorization from both technical and economic standpoints:

1. Elucidate the lignin degradation pathways. Although a variety of lignin-degrading bacteria have been identified using the selective cultivation, their lignin-degrading abilities are still not fully elucidated. Therefore, it is a pressing need to decipher the natural existing but unelucidated lignin degradation pathways. By combining the biodegradation and the biosynthesis process, it will enable a robust biorefinery process of lignocellulose valorization of underutilized aromatics in lignin.
2. Use synthetic biology methods to improve the lignin utilization rate. Most lignin-utilizing bacteria are only able to assimilate a limited range of lignin-derived compounds. To improve the utilization of lignin or lignocellulosic biomass, it is necessary to enhance the related metabolic pathways in these bacteria. However, modifying the metabolic pathways of naturally existing lignin-utilizing bacteria is challenging, due to the lack of efficient genetic manipulation tools. Developing efficient genome editing tools in these native lignin-utilizing bacteria is necessary. Alternatively, by gaining more insights into the way that bacteria break down lignin, it is possible to use design–build–test–learn (DBTL) cycle to construct more efficient lignin utilization pathways in model microorganisms via synthetic biology principles.
3. To accomplish the synthesis of complex phenolic compounds, advanced technologies such as enzyme modification and protein engineering could be employed to improve rate-limiting enzymes with the higher catalytic selectivity and efficiency. In addition, more S-type lignin-derived pathways should be explored to take the full advantage of lignin valorization for diversiform aromatic products.

In summary, the effective use of lignin can drastically increase the cost-effectiveness of the lignocellulosic biorefinery, leading to a more sustainable and circular economy. Despite that substantial advances have been achieved in lignin utilization in recent decades, more endeavors are needed to generate high-value compounds from lignin. The research focusing on microbially mediated lignin valorization is gaining the momentum due to its low energy requirements and environmental benefits, making it an essential part of bioconversion processes. Exploring the process of lignin breaking down provides avenues for leveraging the metabolic engineering to convert lignin into valuable products. To achieve the productive valorization of lignin and the synthesis of value-added products, further research is necessary in the realms of lignin depolymerization, microbial metabolic engineering, and industrial process scale-up.
